# Bis(2,6-dimethyl­pyridinium) tetra­bromido­cobaltate(II)

**DOI:** 10.1107/S160053680800439X

**Published:** 2008-02-20

**Authors:** Basem Fares Ali, Rawhi H. Al-Far, Salim F. Haddad

**Affiliations:** a Department of Chemistry, Al al-Bayt University, Mafraq 25113, Jordan; b Faculty of Information Technology and Science, Al-Balqa’a Applied University, Salt, Jordan; c Department of Chemistry, The University of Jordan, Amman, Jordan

## Abstract

In the crystal structure of the title compound, (C_7_H_10_N)_2_[CoBr_4_], the [CoBr_4_]^2−^ anion is connected to two cations through N—H⋯Br and H_2_C—H⋯Br hydrogen bonds to form two-dimensional cation–anion–cation layers normal to the crystallographic *b* axis. Inter­actions of the π–π type are absent between cations in the stacks [centroid–centroid separation = 5.01 (5) Å]. Significant inter­molecular Br–aryl inter­actions are present in the structure, especially an unusually short Br–ring centroid inter­action of 3.78 (1) Å. The coordination geometry of the anion is approximately tetrahedral and a twofold rotation axis passes through the Co atom.

## Related literature

For general background, see: Al-Far & Ali (2007*a* ▶,*b* ▶); Ali & Al-Far (2007 ▶); Allen *et al.* (1997 ▶); Desiraju & Steiner (1999 ▶); Dolling *et al.* (2001 ▶); Hunter (1994 ▶); Panunto *et al.* (1987 ▶); Robinson *et al.* (2000 ▶). For related literature, see: Al-Far & Ali (2008 ▶); Ali & Al-Far (2008 ▶); Allen *et al.* (1987 ▶); Desiraju (1997 ▶); Zhang *et al.* (2005 ▶).
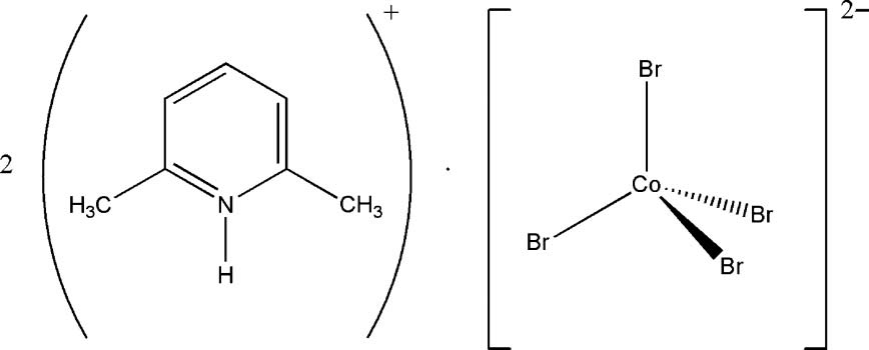


## Experimental

### 

#### Crystal data

(C_7_H_10_N)_2_[CoBr_4_]
*M* *_r_* = 594.89
Orthorhombic, 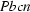

*a* = 17.234 (2) Å
*b* = 9.0691 (10) Å
*c* = 13.729 (2) Å
*V* = 2145.7 (5) Å^3^
*Z* = 4
Mo *K*α radiation
μ = 8.24 mm^−1^
*T* = 293 (2) K
0.40 × 0.30 × 0.20 mm

#### Data collection

Bruker *P*4 diffractometer
Absorption correction: ψ scan;(North *et al.*, 1968 ▶) *T*_min_ = 0.064, *T*_max_ = 0.192
2534 measured reflections
1930 independent reflections
885 reflections with *I* > 2σ(*I*)
*R*_int_ = 0.072

#### Refinement

*R*[*F* ^2^ > 2σ(*F* ^2^)] = 0.055
*wR*(*F* ^2^) = 0.117
*S* = 0.97
1930 reflections
97 parameters
H-atom parameters constrained
Δρ_max_ = 0.55 e Å^−3^
Δρ_min_ = −0.40 e Å^−3^

### 

Data collection: *XSCANS* (Siemens, 1996 ▶); cell refinement: *XSCANS*; data reduction: *SHELXTL* (Sheldrick, 2008 ▶); program(s) used to solve structure: *SHELXS97* (Sheldrick, 2008 ▶); program(s) used to refine structure: *SHELXL97* (Sheldrick, 2008 ▶); molecular graphics: *SHELXTL*; software used to prepare material for publication: *SHELXTL*.

## Supplementary Material

Crystal structure: contains datablocks I, global. DOI: 10.1107/S160053680800439X/at2543sup1.cif

Structure factors: contains datablocks I. DOI: 10.1107/S160053680800439X/at2543Isup2.hkl

Additional supplementary materials:  crystallographic information; 3D view; checkCIF report

## Figures and Tables

**Table:** 

Br1—Co1	2.4002 (13)
Co1—Br2	2.4044 (15)

**Table:** 

Br1—Co1—Br1^i^	115.28 (9)
Br1—Co1—Br2	108.06 (3)
Br1—Co1—Br2^i^	108.37 (4)
Br2—Co1—Br2^i^	108.54 (9)

Symmetry code: (i) 
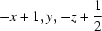
.

**Table 2:** Hydrogen-bond geometry (Å, °)

*D*—H⋯*A*	*D*—H	H⋯*A*	*D*⋯*A*	*D*—H⋯*A*
N1—H1⋯Br2	0.86	2.51	3.366 (7)	176
C7—H7*A*⋯Br2	0.96	2.97	3.856 (10)	153

**Fig. 1.**
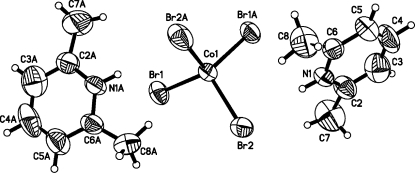
A view of the asymmetric unit of (I), with the atom-numbering scheme. Displacement ellipsoids are drawn at the 50% probability level. [Symmetry operation: (A) -x + 1, y, -z + 1/2].

**Fig. 2.**
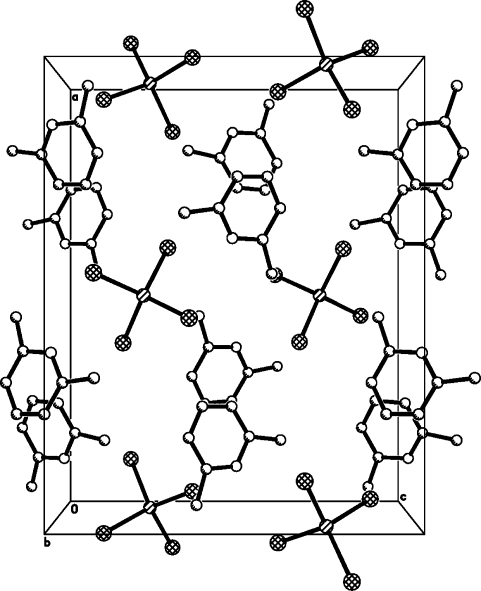
A packing diagram of (I), shows alternating stacks of anions and cations. Hydrogen atoms have been omitted for clarity.

**Fig. 3.**
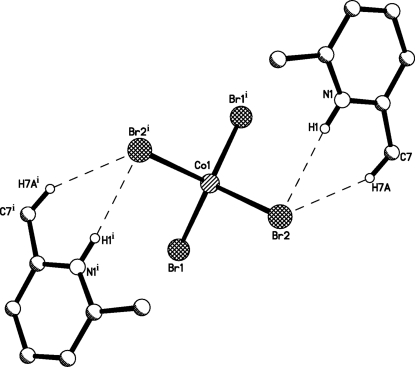
Anion···cation intermolecular interactions. C,N—H···Br—Co intermolecular interactions are shown as dashed lines. Hydrogen atoms not involved in hydrogen bonding omitted for clarity. [Symmetry operation: (i) -x + 1, y, -z + 1/2]

## supplementary crystallographic information

### Comment

Noncovalent interactions play an important role in organizing structural units in both natural and artificial systems (Desiraju, 1997). They exercise important effects on the organization and properties of many materials in areas such as biology (Hunter 1994; Desiraju & Steiner 1999), crystal engineering (see for example: Allen *et al.*,1997; Dolling *et al.*, 2001) and material science (Panunto *et al.*, 1987; Robinson *et al.*, 2000). The interactions governing the crystal organization are expected to affect the packing and then the specific properties of solids. In connection with ongoing studies (Ali & Al-Far, 2008; Al-Far & Ali, 2008; Ali & Al-Far, 2007; Al-Far & Ali, 2007*a*,*b*) of the structural aspects of halo-metal anion salts, we herein report the crystal structure of the title compound (I) along with its crystal supramolecularity.

The asymmetric unit in (I), contains half an anion and one cation (Fig. 1). The geometry of CoBr_4_^2-^ anions is nearly tetrahedral (*T_d_*) about Co metal (Table 1). Co—Br distances are similar, but Co—Br that are engaged in Co—Br···H—N,*C* hydrogen bonding, Co—Br2 and Co—Br2 [1 - *x*, *y*, 1/2 - *z*], are slightly longer than the others (Table 1). The bond distances and angles fall in the range of those reported previously for compounds containing Co—Br anions (Ali & Al-Far 2008; Al-Far & Ali 2008; Zhang *et al.*, 2005). In the cation, the bond lengths and angles are within normal range (Allen *et al.*, 1987).

The packing of the structure (Fig. 2) can be regarded as alternating stacks of anions and stacks of cations. The anion stacks are parallel to the cation stacks, with Co···Co distance of 9.0691 (10) Å (*b* axis), with no significant inter- and intra-stack halogen···halogen interactions (shortest Br···Br interactions being 4.4236 (20) Å). The anions and cations are interacting significantly through extensive N—H···Br and C—H···Br hydrogen bonding involving Br^-^ anions and N—H and CH_3_ groups (Table 2; Fig. 3). These interactions link anions and cations into two-dimensional cation···anion···cation layers approximately normal to the crystallographic *b* axis (Fig. 3).

There is no π···π stacking of cations, the inter-stack centroid separations X1A···X1A [1 - *x*, *y*, 1/2 - *z*] and X1A···X1A [3/2 - *x*, 1/2 + *y*, *z*] being 5.01 (5) Å. This correlates well with the significant intermolecular Br···aryl interactions present in the structure. These are represented by the unusually short Br2···X1A [1 - *x*, -*y*, 1 - *z*] contact (3.78 (1) Å) and Br1···X1A [1 - *x*, *y*, 1/2 - *z*] (4.17 (3) Å) interaction.

### Experimental

Boiling CoCl_2_(1.0 mmol), dissolved in absolute ethanol (10 ml) was added to a stirred absolute ethanol solution (10 ml) of 2,6-lutidine (1 mmol) and 48% HBr (3 ml). The mixture was then treated with liquid Br_2_ (2 ml). After refluxing for *ca* 1 h, the mixture was filtered off and allowed to evaporate undisturbed at room temperature. The salt crystallized out over 1 d as blue crystals.

### Refinement

H atoms bound to carbon and nitrogen were placed at idealized positions [C—H = 0.93 and 0.96 Å and N—H = 0.86 Å] and allowed to ride on their parent atoms with *U*_iso_ fixed at 1.2 or 1.5 *U*_eq_(C,*N*).

### Crystal data

**Table:**

(C_7_H_10_N)_2_[CoBr_4_]	*F*_000_ = 1140
*M**_r_* = 594.89	*D*_x_ = 1.842 Mg m^−^^3^
Orthorhombic, *P**b**c**n*	Mo *K*α radiation λ = 0.71073 Å
Hall symbol: -P 2n 2ab	Cell parameters from 36 reflections
*a* = 17.234 (2) Å	θ = 2.4–16.8º
*b* = 9.0691 (10) Å	µ = 8.24 mm^−^^1^
*c* = 13.729 (2) Å	*T* = 293 (2) K
*V* = 2145.7 (5) Å^3^	Chunk, blue
*Z* = 4	0.40 × 0.30 × 0.20 mm

### Data collection

**Table:**

Bruker P4 diffractometer	1930 independent reflections
Radiation source: fine-focus sealed tube	885 reflections with *I* > 2σ(*I*)
Monochromator: graphite	*R*_int_ = 0.072
Detector resolution: 3 pixels mm^-1^	θ_max_ = 25.2º
*T* = 293(2) K	θ_min_ = 2.4º
ω Scans scans	*h* = −1→20
Absorption correction: ψ scan(North *et al.*, 1968)	*k* = −1→10
*T*_min_ = 0.064, *T*_max_ = 0.192	*l* = −16→1
2534 measured reflections	

### Refinement

**Table:**

Refinement on *F*^2^	Hydrogen site location: inferred from neighbouring sites
Least-squares matrix: full	H-atom parameters constrained
*R*[*F*^2^ > 2σ(*F*^2^)] = 0.055	*w* = 1/[σ^2^(*F*_o_^2^) + (0.0321*P*)^2^] where *P* = (*F*_o_^2^ + 2*F*_c_^2^)/3
*wR*(*F*^2^) = 0.117	(Δ/σ)_max_ < 0.001
*S* = 0.97	Δρ_max_ = 0.55 e Å^−^^3^
1930 reflections	Δρ_min_ = −0.40 e Å^−^^3^
97 parameters	Extinction correction: SHELXL97, Fc^*^=kFc[1+0.001xFc^2^λ^3^/sin(2θ)]^-1/4^
Primary atom site location: structure-invariant direct methods	Extinction coefficient: 0.0060 (4)
Secondary atom site location: difference Fourier map	

### Special details

**Table:**

Geometry. All e.s.d.'s (except the e.s.d. in the dihedral angle between two l.s. planes) are estimated using the full covariance matrix. The cell e.s.d.'s are taken into account individually in the estimation of e.s.d.'s in distances, angles and torsion angles; correlations between e.s.d.'s in cell parameters are only used when they are defined by crystal symmetry. An approximate (isotropic) treatment of cell e.s.d.'s is used for estimating e.s.d.'s involving l.s. planes.
Refinement. Refinement of *F*^2^ against ALL reflections. The weighted *R*-factor *wR* and goodness of fit *S* are based on *F*^2^, conventional *R*-factors *R* are based on *F*, with *F* set to zero for negative *F*^2^. The threshold expression of *F*^2^ > σ(*F*^2^) is used only for calculating *R*-factors(gt) *etc*. and is not relevant to the choice of reflections for refinement. *R*-factors based on *F*^2^ are statistically about twice as large as those based on *F*, and *R*- factors based on ALL data will be even larger.

### Fractional atomic coordinates and isotropic or equivalent isotropic displacement parameters (Å^2^)

**Table:**

	*x*	*y*	*z*	*U*_iso_*/*U*_eq_	
Br1	0.39353 (5)	−0.42729 (12)	0.18719 (7)	0.0680 (4)	
Co1	0.5000	−0.2856 (2)	0.2500	0.0500 (6)	
Br2	0.45145 (6)	−0.13081 (14)	0.37845 (7)	0.0845 (5)	
N1	0.6230 (4)	−0.1559 (9)	0.4945 (5)	0.056 (2)	
H1	0.5806	−0.1498	0.4618	0.067*	
C2	0.6211 (6)	−0.2366 (13)	0.5769 (8)	0.071 (3)	
C3	0.6900 (8)	−0.2499 (13)	0.6250 (9)	0.097 (4)	
H3	0.6928	−0.3052	0.6819	0.116*	
C4	0.7556 (7)	−0.1811 (15)	0.5891 (10)	0.099 (4)	
H4	0.8025	−0.1918	0.6218	0.119*	
C5	0.7527 (6)	−0.0991 (13)	0.5078 (8)	0.085 (4)	
H5	0.7973	−0.0526	0.4852	0.102*	
C6	0.6850 (6)	−0.0840 (11)	0.4588 (6)	0.061 (3)	
C7	0.5478 (6)	−0.3092 (14)	0.6029 (8)	0.116 (5)	
H7A	0.5083	−0.2813	0.5572	0.174*	
H7B	0.5325	−0.2795	0.6672	0.174*	
H7C	0.5546	−0.4142	0.6012	0.174*	
C8	0.6727 (5)	0.0072 (13)	0.3689 (7)	0.106 (4)	
H8A	0.6197	−0.0011	0.3483	0.159*	
H8B	0.7063	−0.0274	0.3180	0.159*	
H8C	0.6843	0.1086	0.3829	0.159*	

### Atomic displacement parameters (Å^2^)

**Table:**

	*U*^11^	*U*^22^	*U*^33^	*U*^12^	*U*^13^	*U*^23^
Br1	0.0521 (6)	0.0757 (8)	0.0762 (7)	−0.0135 (6)	−0.0140 (6)	0.0045 (6)
Co1	0.0379 (9)	0.0594 (14)	0.0527 (10)	0.000	−0.0008 (9)	0.000
Br2	0.0537 (6)	0.1130 (11)	0.0867 (8)	0.0146 (7)	−0.0040 (6)	−0.0405 (7)
N1	0.036 (4)	0.069 (6)	0.063 (5)	−0.006 (4)	0.001 (4)	−0.005 (5)
C2	0.056 (7)	0.081 (8)	0.075 (7)	−0.007 (6)	−0.003 (6)	0.018 (7)
C3	0.106 (10)	0.080 (10)	0.103 (9)	0.013 (8)	−0.024 (9)	0.009 (8)
C4	0.065 (8)	0.116 (12)	0.117 (11)	0.032 (9)	−0.045 (8)	−0.020 (9)
C5	0.055 (7)	0.113 (11)	0.087 (8)	0.006 (7)	−0.012 (7)	−0.007 (8)
C6	0.050 (6)	0.068 (7)	0.065 (7)	−0.014 (6)	0.005 (5)	−0.017 (6)
C7	0.104 (10)	0.124 (12)	0.120 (9)	−0.034 (9)	0.006 (8)	0.055 (9)
C8	0.085 (8)	0.150 (13)	0.082 (8)	−0.045 (9)	0.002 (7)	0.040 (9)

### Geometric parameters (Å, °)

**Table:**

Br1—Co1	2.4002 (13)	C4—C5	1.341 (15)
Co1—Br1^i^	2.4002 (13)	C4—H4	0.9300
Co1—Br2	2.4044 (15)	C5—C6	1.354 (12)
Co1—Br2^i^	2.4044 (15)	C5—H5	0.9300
N1—C2	1.348 (11)	C6—C8	1.501 (12)
N1—C6	1.345 (10)	C7—H7A	0.9600
N1—H1	0.8600	C7—H7B	0.9600
C2—C3	1.363 (13)	C7—H7C	0.9600
C2—C7	1.468 (12)	C8—H8A	0.9600
C3—C4	1.383 (15)	C8—H8B	0.9600
C3—H3	0.9300	C8—H8C	0.9600
			
Br1—Co1—Br1^i^	115.28 (9)	C4—C5—C6	120.1 (12)
Br1—Co1—Br2	108.06 (3)	C4—C5—H5	119.9
Br1^i^—Co1—Br2	108.37 (4)	C6—C5—H5	119.9
Br1—Co1—Br2^i^	108.37 (4)	N1—C6—C5	117.0 (10)
Br1^i^—Co1—Br2^i^	108.06 (3)	N1—C6—C8	117.1 (9)
Br2—Co1—Br2^i^	108.54 (9)	C5—C6—C8	125.9 (10)
C2—N1—C6	126.0 (8)	C2—C7—H7A	109.5
C2—N1—H1	117.0	C2—C7—H7B	109.5
C6—N1—H1	117.0	H7A—C7—H7B	109.5
N1—C2—C3	115.7 (10)	C2—C7—H7C	109.5
N1—C2—C7	117.9 (9)	H7A—C7—H7C	109.5
C3—C2—C7	126.3 (11)	H7B—C7—H7C	109.5
C2—C3—C4	120.0 (11)	C6—C8—H8A	109.5
C2—C3—H3	120.0	C6—C8—H8B	109.5
C4—C3—H3	120.0	H8A—C8—H8B	109.5
C5—C4—C3	121.1 (11)	C6—C8—H8C	109.5
C5—C4—H4	119.5	H8A—C8—H8C	109.5
C3—C4—H4	119.5	H8B—C8—H8C	109.5
			
C6—N1—C2—C3	−2.9 (16)	C3—C4—C5—C6	−1(2)
C6—N1—C2—C7	−178.9 (10)	C2—N1—C6—C5	3.0 (15)
N1—C2—C3—C4	0.8 (18)	C2—N1—C6—C8	−176.3 (9)
C7—C2—C3—C4	176.5 (12)	C4—C5—C6—N1	−1.0 (16)
C2—C3—C4—C5	1(2)	C4—C5—C6—C8	178.2 (11)

Symmetry codes: (i) −*x*+1, *y*, −*z*+1/2.

### Hydrogen-bond geometry (Å, °)

**Table:**

*D*—H···*A*	*D*—H	H···*A*	*D*···*A*	*D*—H···*A*
N1—H1···Br2	0.86	2.51	3.366 (7)	176
C7—H7A···Br2	0.96	2.97	3.856 (10)	153
